# Radiation-Induced High-Temperature Conversion of Cellulose

**DOI:** 10.3390/molecules191016877

**Published:** 2014-10-21

**Authors:** Alexander V. Ponomarev, Boris G. Ershov

**Affiliations:** A.N. Frumkin Institute of Physical Chemistry and Electrochemistry, Russian Academy of Sciences, Leninsky prosp. 31, Moscow 119991, Russia; E-Mail: ershov@ipc.rssi.ru

**Keywords:** cellulose, radiolysis, electron-beam distillation, glucopyranose, macroradical, chain decomposition, furfural, alternative fuel

## Abstract

Thermal decomposition of cellulose can be upgraded by means of an electron-beam irradiation to produce valuable organic products via chain mechanisms. The samples being irradiated decompose effectively at temperatures below the threshold of pyrolysis inception. Cellulose decomposition resembles local “explosion” of the glucopyranose unit when fast elimination of carbon dioxide and water precede formation of residual carbonyl or carboxyl compounds. The dry distillation being performed during an irradiation gives a liquid condensate where furfural and its derivatives are dominant components. Excessively fast heating is adverse, as it results in a decrease of the yield of key organic products because pyrolysis predominates over the radiolytic-controlled decomposition of feedstock. Most likely, conversion of cellulose starts via radiolytic formation of macroradicals do not conform with each other, resulting in instability of the macroradical. As a consequence, glucosidic bond cleavage, elimination of light fragments (water, carbon oxides, formaldehyde, *etc.*) and formation of furfural take place.

## 1. Introduction

Cellulose, which is a polymer with repeating glucose units, contains a lot of carbon and hydrogen atoms, is of great interest as a possible renewable raw material for production of organic reagents and fuels. Technological and laboratory processes that include radiation treatment of cellulose are well-known [1,2,3]. Examples are sterilization of medicines and bandaging materials; decomposition of cellulose feedstock to intensify its hydrolytic processing into food, forage and technical products; production of explosives, lacquers, viscose, various monoses and feed for farm animals; radiation preprocessing of cellulose for production of its esters (nitrates, xanthates, *etc.*) and so on. The cellulose macromolecule length decreases during the process as the absorbed dose increases. Simultaneously the physical and chemical properties of the material are changing—solubility is increasing, mechanical strength is dropping, hydrolytic cellulose-to-glucose conversion is accelerating, *etc.* [2,3,4,5,6,7,8]. It is also important to emphasize that cellulose and its derivatives represent convenient models for the analysis of the effects of irradiation on biological systems and the geochemical degradation of biomass. All these issues have stimulated interest in the study of mechanisms of the radiolytic transformation of cellulose and elucidation of the effects of temperature, feedstock characteristics (composition, state) and other factors on the efficiency of radiation decomposition of plant materials [2,9,10,11].

Initially, studies of cellulose decomposition with cleavage of the polymer chain were mainly carried out at room and moderate temperatures when pyrolytic processes related to feedstock distillation do not occur. The situation has changed considerably in the past few years when it was shown [12,13] that the electron-beam radiolysis of cellulose at higher temperatures (>190 °C) results in deeper transformations with formation of liquid organic products and gases.

At present, electron accelerators are used most widely as radiation sources due to economic and ecological reasons [3]. The cost of irradiation by accelerated electrons is significantly lower than by γ-quanta. In addition, the use of radioactive isotopes is associated with the risk of their emission to the environment, whereas electron accelerators are environmentally safe. The electron beam can be produced only in the case when the accelerator is powered up. An additional substantial advantage of electron accelerators is the possibility of their use as heaters for combined radiation-thermal treatment. If high absorbed dose rates are used, the accelerated electrons not only initiate chemical processes in the bulk of the irradiated material, but also heat it. The effect of dose rate on cellulose decomposition is considered in this work.

The dual action of an electron beam (ionization and heating) can be applied to upgrade the dry distillation method (M0)—high-temperature decomposition in air-free conditions [1]. In this method heating provides both formation of decomposition products and their distillation off. Dry distillation (or slow pyrolysis), being a method of deep decomposition, converts cellulose mainly to СО_2_, Н_2_О and charred residue. The yield of desirable organic products is low. Radiolysis also belongs to the decomposition methods. In contrast to pyrolysis, radiolysis originates via high-energy ionization and excitation followed by reactions of short-lived radicals and ions. Lower thermal stability of radiation-induced intermediates could affect both the mechanism of high-temperature transformations of cellulose and the assortment of final products. Combination of radiolysis and slow pyrolysis (pyrogenic distillation) is studied in this work to reveal new prospects for converting renewable cellulose biomass into the demanded products.

**Figure 1:**
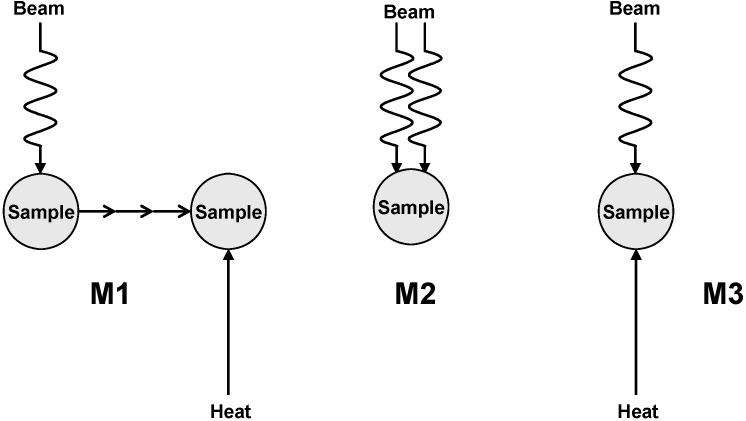
Combinations of electron-beam treatment and heating.

Three accessible combinations of irradiation and heating were considered (see Figure 1). The first mode (М1) provided two-stage treatment: initially the sample was irradiated at ambient temperature (~20 °C) by a low-current electron beam (to avoid a radiation heating), and then the irradiated sample was heated up by a conventional way to the temperature providing dry distillation. The second mode (М2) provided single-stage treatment: the powerful electron beam performs simultaneously both an irradiation and a heating to the temperature initiating distillation. As a result, both radiation-induced chemical and thermal processes proceed in parallel on exposure to an electron beam, resulting in transformation of cellulose samples into stable liquid organic products in a single-stage process. The third mode (М3) provided heating of the sample by two sources simultaneously: by both a low-current electron beam and a conventional heater.

## 2. Decomposition of Cellulose at Moderate Absorbed Dose Rates

### 2.1. Regularities and Products of Cellulose Decomposition

**Figure 2:**
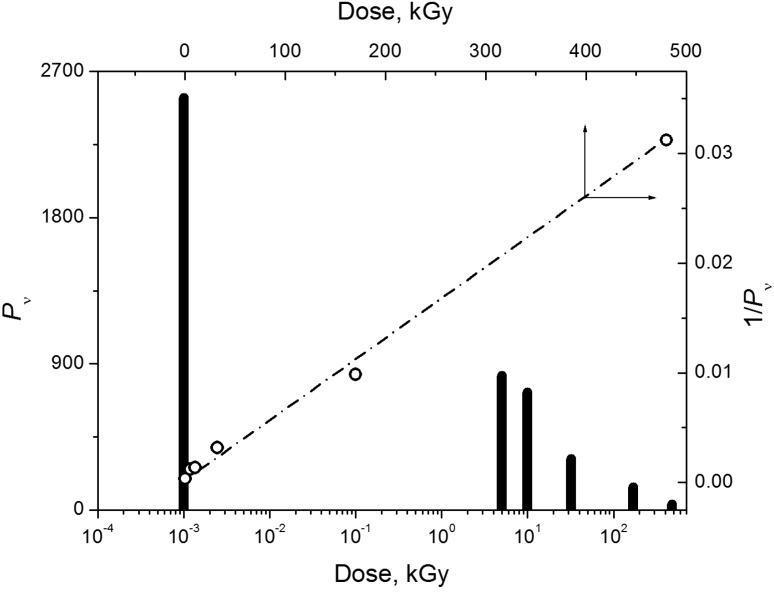
Dose effect on degree of polymerization of cotton cellulose [3].

The action of ionizing radiation on cellulose, as on other polysaccharides, mainly results in its decomposition. This process changes all the physicochemical properties of the material: its structural state, mechanical strength, solubility in various fluids, reactivity and so on. The change in the degree of polymerization of cotton cellulose (*P_v_* is the average number of units in the polymer chain) with the absorbed dose of γ-radiation is shown in Figure 2.

It can be seen that the degree of polymerization, which reached ≈2.5 thousand units in the initial material, decreased to almost 30 after 480 kGy (1 kGy = 1 kJ/kg) irradiation, *i.e.*, the average chain length decreased by a factor of approximately 80. The material was extensively destroyed. The decomposition process is adequately described by equations based on random cleavages of a polymeric chain [2]. The dependence of the degree of polymerization of cellulose (*P*) on the dose (at *D* ≤ 200 kGy) is linear and obeys Equation (1) (see Figure 2):

(1) $$\frac{1}{P}+\frac{1}{P^{0}}=\frac{1.62G^{0}D}{N_{A}k}$$

where *P*^0^ is the initial degree of polymerization of cellulose, *k* is an auxiliary coefficient, *N_A_* is the Avogadro number.

The radiation-chemical yield of the decomposition (below called the decomposition yield, *G*^0^) is 6 bonds per 100 eV of the absorbed energy (~0.6 μmol/J), it is almost independent of the γ-radiation or accelerated electrons dose rate, on the starting degree of polymerization or microcrystallinity of the initial celluloses, or on whether the irradiation was carried out in vacuum, under inert gases or in air. The lack of any visible effect of air on the efficiency of cellulose decomposition is a result of the dense crystal packing of cellulose, which hampers the diffusion of oxygen to reaction centres.

Application of higher doses (ε200 kGy) reduces the rate of decomposition. This effect is caused by accumulation of decomposition products in the material. Intermediates of radiation chemical transformations and final radiolysis products capture electrons, radicals and excitation energy, thus retarding further degradation of the biopolymer. For very high doses (>1 MGy), the cellulose decomposition may be described by empirical equation (1), in which the *D* parameter is replaced by *D^q^*, where exponent *q* is equal to 0.83 [14,15] or 0.73 [16]. Oxygen participates only in the surface radiolytic oxidation, resulting in the formation of carbonyl and carboxyl groups on the polymer surface. Decomposition of cellulose becomes much slower upon moistening [17,18].

Radiation at room temperature at low dose rates leads to deep chemical transformations of cellulose [19,20]. Among water-soluble products, cellobiose, glucose, arabinose, glyoxal and 2-ketogluconic and some other acids were detected. Hydrolysis of the water-soluble fraction results in the formation of xylose, arabinose, glucuronic and formic acids, malondialdehyde, *etc.*

**Figure 3:**
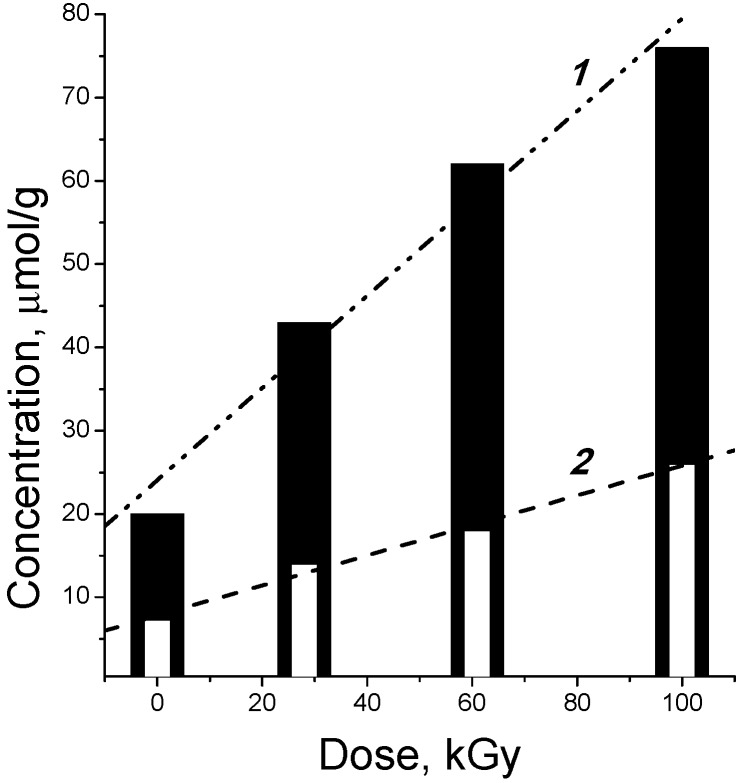
Accumulation of carbonyl (1) and carboxyl (2) groups during radiolysis of cotton cellulose [18].

The effect of irradiation at room temperature on the concentration of the carbonyl and carboxyl groups in the cotton cellulose is illustrated in Figure 3. The data show that the cellulose decomposition gives mainly carbonyl compounds. The carbonyl group yield is equal to 6.5 ± 0.5 per 100 eV of absorbed energy, whereas that of carboxyl groups ranges from 0.9 to 1.8 [16,21,22,23,24,25]. Radiolytic formation of both carbonyl and carboxyl groups is almost independent of their starting concentration in the initial sample, and is determined by the absorbed dose. For instance, ~3 mmol of C=O and 1 mmol of CO_2_H groups are formed in 100 g of cellulose at 50 kGy. The type of cellulose and the initial concentration of carbonyl and carboxyl compounds in it do not affect the product yields noticeably (Table 1).

**Table 1:** Initial concentrations and radiation chemical yields of oxygen-containing groups upon the cellulose γ-radiolysis [2].

Cellulose type	Concentration, mmol/100 g		Yield, mmol/100 eV	
	-CHO	-CO_2_H	-CHO	-CO_2_H
Sulfite cellulose	0.13	0.24	6.0	2.0
High-viscosity sulfite cellulose	0.53	3.16	5.4	1.8
Cotton cellulose	0.45	0.45	5.9	2.5

The concentrations of the carbonyl and carboxyl groups at high radiation doses are described by the non-linear dependence:

*c = BD^q^* (2)

where coefficient *B* is 6.8 × 10^−7^ for carbonyl groups and 1.8 × 10^−7^ for carboxyl groups [14], the exponent *q* is equal to 0.50 ± 0.69 for both groups [16,21]. It is obvious that upon cellulose decomposition at high absorbed doses, carbonyl and carboxyl compounds are formed and then involved in subsequent radiolytic transformations.

Carbon monoxide, carbon dioxide and hydrogen are the major volatile products of cellulose radiolysis [22,26,27,28,29]. Their yields under vacuum at doses of ~300 kGy are 6.0, 0.9 and 3.2 molecules per 100 eV of absorbed energy, respectively. Methane was also found among the volatile products.

### 2.2. The Decomposition Mechanism

According to the generally accepted ideas about the nature of free radicals [18,30,31,32,33,34,35,36], the radical states (unpaired electrons) in irradiated cellulose are predominantly located in positions 1 and 4 of the glucopyranose rings of the polymer. The probabilities of the C(1)-H and C(4)-H bond cleavages are rather similar at the liquid nitrogen temperature. Irradiated samples of frozen cellulose are dark-blue coloured due to optical absorption of electrons captured by cellulose. Wet samples have more intense colour.

The radiolytic decomposition of cellulose (RH) is based on ionization, neutralization of ion-electron pairs and decay of excited states:

RH → RH^+^ + e^−^ (3)

RH^+^ + e^−^ → RH* (4)

RH* → R^•^ +•H^•^ (5)

The excited states mainly decay with cleavage of the C(1)-H or C(4)-H bonds in the glucopyranose rings to give the R• radicals (no RO• radicals were detected in the irradiated cellulose samples). Free hydrogen atoms abstract glucopyranose hydrogen atoms giving the same R• radicals:

RH + •H^•^→ R^•^ + H_2_ (6)

**Scheme 1:**
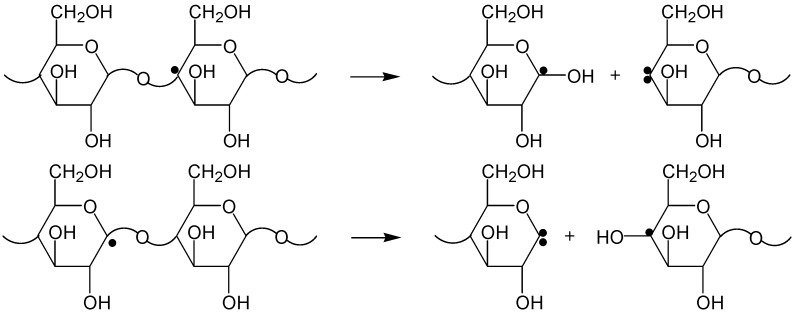
Decomposition of primary radicals.

The radiation chemical event in the cellulose polymeric chain occurs as a local 'explosion' of the glucopyranose ring. Decomposition of the ring is a consequence of the fact that cellulose is a rigid chain polymer, and the metastable radical centre formed upon its irradiation can be transformed into a stable radical only if the polymeric chain is cleaved and the monomeric (glucopyranose) unit is destroyed. The primary radicals R• are unstable due to substantial deformations caused by mismatch between the electron configurations of the radical carbon atom (*sp*^2^ hybridization, planar structure) and the starting macromolecular unit (*sp*^3^ hybridization with the tetrahedral structure). Even at room temperature, the radicals R• dissociate with cleavage of the glycosidic linkage (Scheme 1) [10].

Further decomposition of the fragments formed in these reactions (see Scheme 1) gives stable low-molecular-mass products. Thus, the cleavage of the cellulose polymeric chain results in elimination of a CO_2_ molecule and formation of substances containing carbonyl and carboxyl groups. As noted above, the yields of carbonyl groups and CO_2_ are rather close to the yield of polymeric chain decomposition. This means that each cellulose chain cleavage event is accompanied by formation of a CO_2_ molecule and a carbonyl group.

Decrease in the irradiation temperature from 300 to 77 K results in almost twofold reduction of the cellulose decomposition yield and the yields of carbonyl compounds, CO_2_ and CO. It is important to emphasize that the H_2_ yield remains unchanged. Hence, the process of C-H bonds cleavage is not temperature-dependent, whereas the glucopyranose ring decomposition may be retarded on cooling.

### 2.3. Temperature Effect

**Figure 4:**
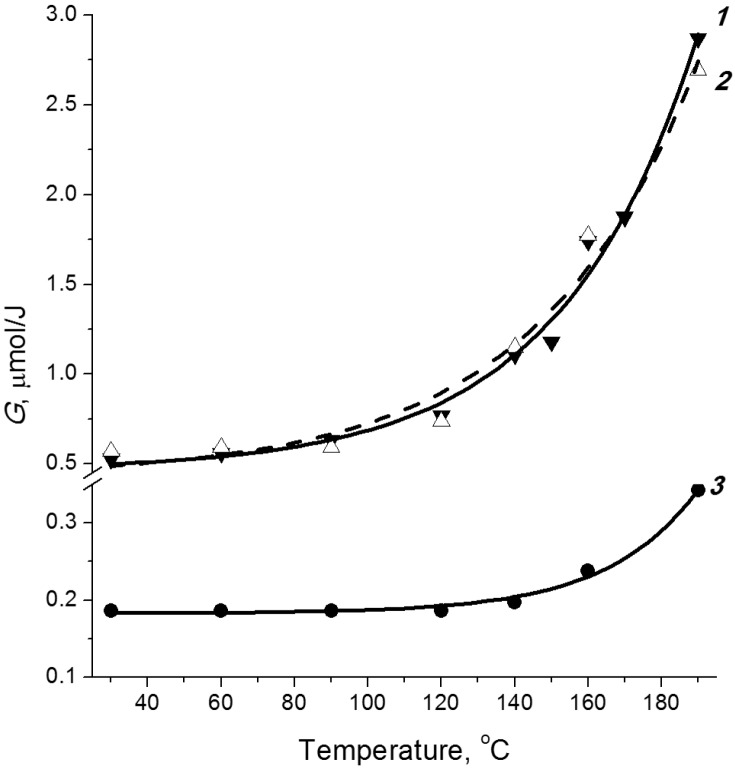
Temperature dependences of cellulose decomposition yield (*1*) and formation of carbonyl (*2*) and carboxyl groups (*3*) [15].

The radiation decomposition of cellulose is a temperature-dependent process [37]. As the temperature at which irradiation is carried out increases, the degree of polymerization rapidly decreases. The temperature effect on the yield of radiation chemical decomposition of cellulose is shown in Figure 4. One can distinguish two temperature ranges differing by the efficiency of the radiation decomposition of the biopolymer. A temperature rise from room temperature to ~100 °C has little effect on the decomposition yield, while higher temperatures result in its significant increase. For example, the efficiency of radiation fragmentation at 190 °C is 6 times higher than at room temperature (see Figure 3).

The temperature effect on the cellulose decomposition is caused by the chain mechanism of this process. The decomposition is initiated by radicals formed upon irradiation. The activation energy of the chain process [37] is 23.4 ± 1.6 kJ/mol. Such a value is typical of radical diffusion in a polymer and corresponds to the energy of conformational oscillations in the glucopyranose unit [38]. An increase in temperature initiates the transfer of the radical centre to a nearby polymeric chain, thus causing its decomposition. Analogous radical chain transfer and decomposition processes further propagate through the polymer bulk. The ratios of the major product yields remain the same as upon irradiation at room temperature (see Figure 4). This fact evidences that the low- and high-temperature processes are the same, being accompanied by decomposition of glucopyranose rings and elimination of CO_2_ to give compounds containing carbonyl and carboxyl groups. The cellulose decomposition at high temperatures is also characterized by increase in the yields of hydrogen and carbon dioxide.

The efficiency of the cellulose decomposition depends on the succession of application of irradiation and heating [39]. Both irradiation and heat treatment decrease the degree of cellulose polymerization and increase the yield of hydrolytic sugar formation. The number of bond cleavages per cellulose macromolecule upon two-stage treatment in the sequence heating→irradiation obeys Equation (7):

*S*_(T→R)_ = *S*_T_ + *S*_R_ (7)

where *S*_R_ is the number of cleavages upon single-stage radiolysis, *S*_T_ is the number of cleavages upon single-stage thermal treatment. Therefore the succession heating→irradiation is characterized by an additive effect. On the other hand, the sequence irradiation→heating results in a superadditive number of cleavages [2]:

*S*_(R→T)_ = *S*_R_ + *S*_T_*+* β*D* (8)

where β is a coefficient reflecting the increase in efficiency of thermal decomposition of the irradiated cellulose at dose *D*. The sequence irradiation→heating also has superadditive effect on the yield of hydrolytic formation of sugar from the treated cellulose [2].

**Figure 5:**
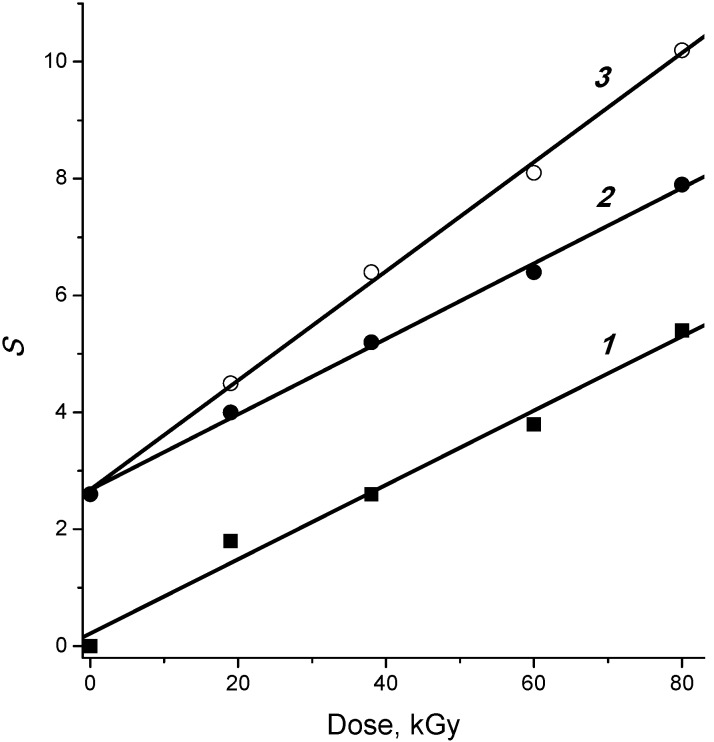
Dependences of the number of bond cleavages (*S*) on the dose for radiolysis of wood cellulose at various combinations of radiation and heating (3 h at 190 °C): (*1*) irradiation, (*2*) heating-irradiation, (*3*) irradiation-heating [2].

The dependences of bond cleavage numbers on the absorbed doses are shown in Figure 5 for some combinations of irradiation and heating procedures. The same slope of straight lines *1* and *2* implies that a preliminary thermal treatment has no effect on the efficiency of the subsequent radiation decomposition. At the same time, the larger slope of line 3 evidences more effective decomposition of cellulose upon the succession irradiation→heating. The coefficient b correlates with the slopes of the dose dependences at the treatment temperature used. At 150, 170 and 190 °C, the values of β are equal to the following bond cleavage numbers per kGy: 0.004, 0.02 and 0.038 [2].

### 2.4. Post-Radiation Distillation (M1 Mode)

The weights of the cellulose samples remained almost unchanged during the course of preliminary irradiation: a decrease in the weights was no greater than 0.1 wt % at absorbed doses *D* to 3 MGy (0.02 kGy/s). The samples remained in a solid state of aggregation; in this case, the microcrystalline cellulose acquired a yellowish nuance.

**Figure 6:**
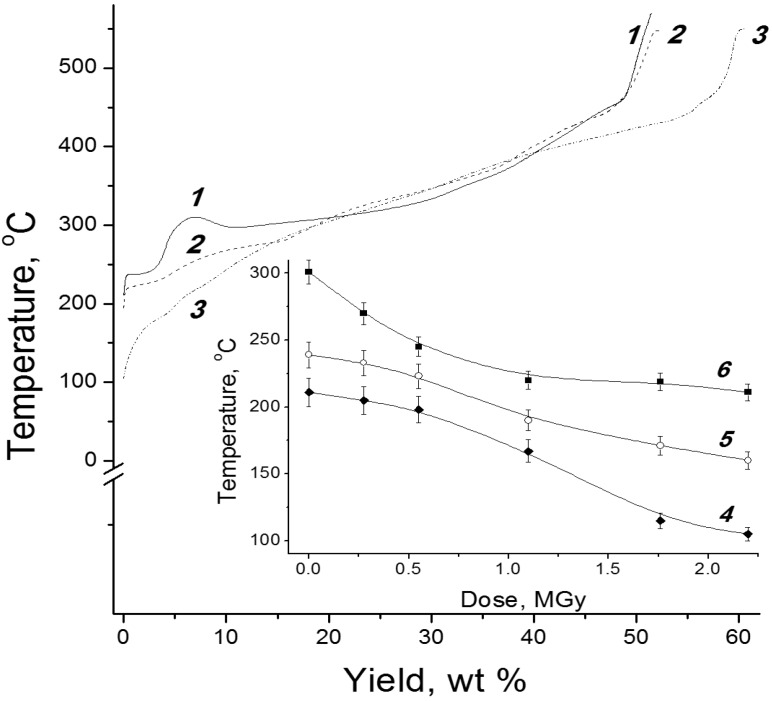
Relationship between the distillation still temperature and the amount of condensate distilled from microcrystalline cellulose at a heater power of 100 W and absorbed doses of (*1*) 0, (*2*) 0.55, and (*3*) 2.2 MGy. Insert: effect of absorbed dose on the temperatures of (*4*) the onset of distillation, (*5*) the appearance of condensate in the receiving tank, and (*6*) the distillation of 10% condensate.

Post-radiation distillation exhibited a number of differences from the ordinary dry distillation of nonirradiated samples [40]. Figure 6 shows that, after irradiation, lower temperatures were required for the appearance of vapors in the distillation still and the appearance of the first drops of a distilled condensate in a receiving tank. At *D* = 2.2 MGy, the overpoints of cellulose decreased by ~100°. Approximately a third of a condensate from cellulose was distilled off after irradiation at lower temperatures.

The appearance of vapors on the distillation of unirradiated cellulose was observed at a temperature of ≈211 °C. A plot of the volume of distilled condensate against the temperature in the distillation still (curve *1* in Figure 6) has a characteristic step, when the volume of the distilled condensate increased, but the temperature did not increase or even temporarily decreased. In this period, water was the main thermolysis product distilled from unirradiated microcrystalline cellulose. Regardless of *D*, a maximum condensate amount was distilled off in a temperature range of 300–370 °С. The differential thermal analysis of distillation revealed intense exothermic peaks between 330 and 350 °C, which suggest the formation and volatilization of gaseous degradation products [41,42].

On the distillation of irradiated cellulose, steps in distillation curves became less pronounced, and they were completely absent at high doses. The first drops of the distilled condensate were colorless: water was also predominant in them. However, the color rapidly changed: the condensate became yellow, and the first 10% of the condensate was brilliant yellow. The intense color of the condensate and the absence of clearly pronounced steps from the distillation curves of the irradiated samples suggest a change in the range of distilled products: heavier organic compounds with different vaporization temperatures appeared. This is also evidenced by an increase in the density of the liquid product as the absorbed dose in the distilled sample was increased.

**Figure 7:**
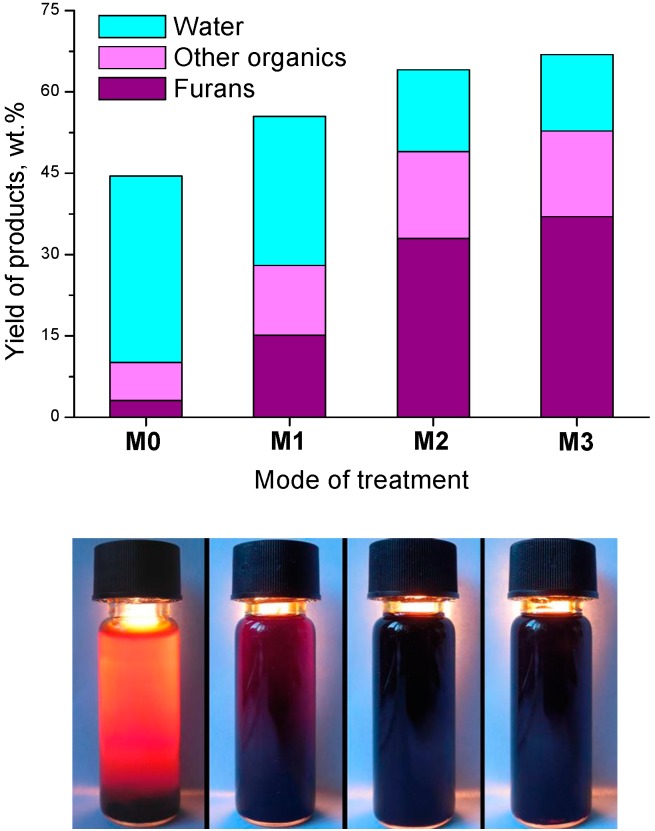
Yields, average fractional composition and photos of condensates, obtained from microcrystalline cellulose (Dose: M1—2 MGy; M2—550 kGy; M3—75 kGy).

Condensates from the irradiated and unirradiated cellulose spontaneously separated into a transparent aqueous organic solution (F1) and a dark opaque tar (F2). The color of F1 from the irradiated samples was much darker (Figure 7). At the same time, F2 from the irradiated cellulose also separated into two phases: the major portion of tar settled at the bottom, and a portion came to the surface. The total tar volume was to 7% on a condensate volume basis. The composition of the bottom (heavier) fraction was only slightly dependent on *D*, and it was generally identical to the composition of tar distilled from unirradiated cellulose. The major portion of this tar consisted of small oligomeric cellulose fragments, which underwent partial dehydration and decarboxylation.

Phase distribution of the distillation products of microcrystalline cellulose changed only slightly: as the absorbed dose *D* was increased to 2.2 MGy, a monotonic decrease in the yield of charcoal by ~7 wt % with a simultaneous increase in the yield of distilled condensate and an almost unchanged yield of gaseous products was observed.

An important change in the component composition of F1 was observed: dissolved furfural and furylmethanol appeared, which were almost absent upon the dry distillation of unirradiated cellulose. At a dose of 2.2 MGy, the yield of furans reached ~22% of the total condensate weight. The composition of the condensate remained almost unchanged as the time interval between irradiation and distillation was increased from several minutes to several days. It is obvious that furans were predominantly formed in the process of the thermolysis of irradiated samples rather than on irradiation: the irradiated cellulose acquired only a slightly yellowish color, which is inconsistent with the observed amount of furans in the condensate. It is likely that, in the course of irradiation, compounds that possess low thermal stability and can be easily converted into furans on subsequent heating were formed.

**Scheme 2:**
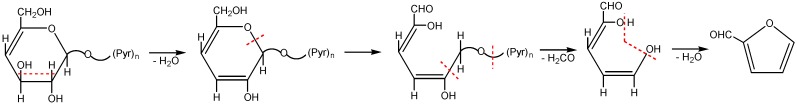
Formation of furfural.

At room temperature, the radicals R• are decomposed according to Scheme 1. The decrease in the length of a polymer chain in cellulose did not cause effects produced by radiolysis: the distillation of the low-molecular-weight analogs of cellulose (cellobiose and cyclodextrins) did not lead to a considerable increase in the yield of furans. Obviously, decomposition products containing new functional groups (carbonyl, carboxyl, and allyl), which were formed in the process of radiolysis, played a key role in the formation of furans. The thermally unstable products of radical recombination or the allyl products of glycoside bond cleavage can be the precursors of furans. For example, furfural can be obtained from the allyl product (from Scheme 1) due to the thermally stimulated elimination of water and decarboxylation (Scheme 2).

The study showed that the preliminary irradiation decreased the initial temperature of the thermal degradation of cellulose. The dry distillation of irradiated cellulose was accompanied by a higher yield of liquid organic compounds, among which the fraction furfural and furylmethanol increased with dose [40].

Irradiation affects differently the post-radiation distillation of cellulose and lignin; this is important from the point of view of wood distillation. Cellulose is characterized by an increase in the yield of liquid organic products, especially, the yield of furfural and its derivatives, with dose. In turn, a decrease in the yield of the liquid organic products of distillation is characteristic of lignin at 1 MGy ≤ *D* ≤ 3 MGy [40]. In this case, the aromatic units of lignin, which play a role of the scavenger of excitation, ions, and radicals, can weaken cellulose degradation in irradiated wood [2,3,43]. This effect was observed in the distillation of irradiated pine sawdust [44]. At *D* = 2 MGy, the yield of the distilled condensate was found to 5.5 wt % lower than that from the unirradiated sawdust; however, the furfural content of the condensate increased (from 5.7 to 11.3 wt %).

## 3. Radiation-Thermal Transformations of Cellulose at High Dose Rates

The use of accelerated electrons at high dose rates makes it possible to implement the direct single-step conversion of cellulose into the liquid condensate (electron-beam distillation mentioned above). It is established [45,46] that at dose rate of ε1.0 kCy/s, such a treatment is accompanied by strong cellulose decomposition, which gives charcoal in addition to the liquid condensate. Decomposition of cellulose via М2 and М3 modes is considered in this part.

### 3.1. Electron-Beam Distillation

**Figure 8:**
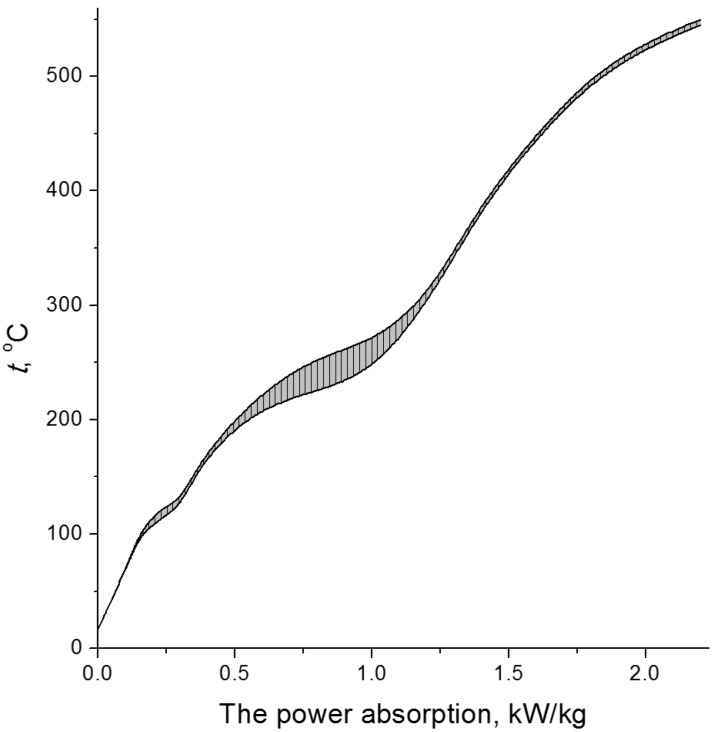
Dynamics of electron-beam heating of cellulose at the dose rate of 2.5 kGy/s (the specific surface area *Z* of the material is 105 cm^2^/g; the volume density is 150 g/dm^3^) [13]. The temperature region of distillation of decomposition products is shaded.

The typical dynamics of electron-beam heating of cellulose at dose rate of 2.5 kGy/s is shown in Figure 8. The most active condensation of vapours in the condenser is observed 3 min after the start of irradiation, whereas within 6 min, vapour generation is completed.

The feebly marked step on the plot at 100 °C is caused by evaporation of the residual water and water formed during radiolysis. In the temperature range of 230–250 °C, the sample decomposes with rapid production of volatile low-molecular-mass products. Then the temperature rise becomes slower (the shaded step on the curve, see Figure 8) due to endothermic evaporation of the decomposition products. The observed yield of condensed products of the cellulose distillation at this stage is ε15 μmol/J, implying that the decomposition proceeds according to the chain mechanism. When the formation of gaseous and liquid products is finished, charcoal is produced in the residue. Further irradiation is accompanied by warming-up of the charred residue.

**Figure 9:**
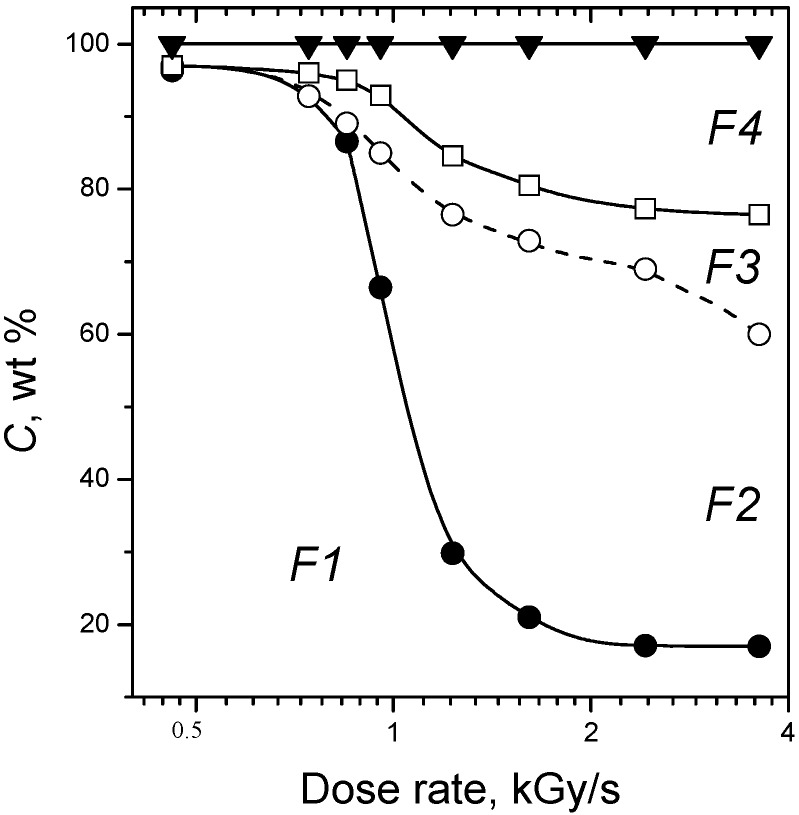
Influence of absorbed dose rate in M2 mode on content *C* of solid (F1), liquid organic (F2), aqueous (F3) and gaseous (F4) fractions in products of microcrystalline cellulose distillation at dose 500 kGy.

Certainly the fraction of decomposition products distilled-off from the irradiation area in M2 mode depends on the temperature of the biopolymer under irradiation. In the study only radiation heating caused by electron beam absorption in the bulk of biopolymer was applied in M2 mode. Accordingly sample heating dynamics was determined by the absorbed electron beam radiation dose rate. The observed phase distribution of cellulose destruction products depending on dose rate is shown on Figure 9. These phase diagrams indicate that efficient decomposition and distillation of biopolymers in M2 mode is observed at dose rates ε1 kGy/s. At dose rates ≤0.6 kGy/s and dose *D* = 500 kGy the weight of irradiated samples of cellulose decreases slightly, mainly as a result of formation and removal of light-end products—H_2_, CO_2_ and CO. At dose rates of 0.6–0.9 kGy/s the distilled-off main product is a colorless homogeneous liquid where water dominates.

The efficiency of the electron-beam conversion of cellulose to liquid organic products was studied depending on the initial temperature and the dispersion degree (specific surface area, *Z*) and the type of starting material [45,46,47]. Cotton cellulose (C1), unbleached pine sulfate cellulose (C2) and bleached pine sulfite cellulose (C3) were chosen for the comparison. Telemetry revealed no noticeable difference in dynamics of the electron-beam distillation of samples C1, C2 and C3 in the pressure range of 730–740 mm Hg in the absence of air. Irradiation with the dose of ~250 kGy is accompanied by formation of heavy fog slowly overflowing from the reactor to the condenser, its colour gradually changing from white to bright-yellow. The condensate is collected in a receiver flask, and its amount increases in the course of irradiation. The vapour formation and production of condensate ended at the dose of ~500 kGy. The condensate is a homogenous odorous brown liquid, its yield amounts to ~60 mass%. The physicochemical characteristics [refractive index (*n*^18^_D_), density (ρ) and relative viscosity (η_0_)] of the condensates obtained from samples C1, C2 and C3 are summarized in Table 2.

**Table 2:** Yield of condensate [*G* (%) of cellulose dry mass] and physicochemical characteristics of condensates (*Z* = 104 cm^2^/g) [45].

Cellulose	*G*	*n*^18^_D_	ρ, kg/dm^3^	η_0_, mPa·s
C1	63	1.4479	1.1639	5.96
C2	58	1.4449	1.1560	5.67
C3	60	1.4455	1.1594	5.79

As mentioned above, a dry black residue (charcoal) remains in the reactor after distillation. Its volume is smaller than that of the starting material, its yield being ~16%–20% of the initial dry cellulose weight. Therefore most of the glucopyranose units are destroyed during the electron-beam distillation.

### 3.2. Products of Electron-Beam Distillation

The major components of the condensates obtained from celluloses C1, C2 and C3 and their relative contents are shown in Table 3. All the condensates have similar composition and include more than 40 organic compounds with molecular masses ranging from 32 to 165 amu. The major liquid products are furans, among which furan-2-carbaldehyde (furfural), 2-furylmethanol, 5-methylfuran-2-carbaldehyde and furan-3-carbaldehyde predominate. The content of water in the condensates is ~8 mass%. Analogous results were obtained earlier [48] in the studies of electron-beam distillation of cotton wool and sulfate cellulose fibres in a gaseous alkane flow. The formation of furfural and other furan derivatives as predominant products of cellulose decomposition upon electron-beam distillation is unexpected because these products are formed in quite low yields upon radiolysis at temperatures below 190 °C (see Section 3) and upon cellulose pyrolysis [49].

**Table 3:** Contents (mass%) of major compounds in the condensates obtained upon radiolysis of cellulose samples (*Z* = 104 cm^2^/g) [45].

Component	Sample		
	C1	C2	C3
Methylformate	0.90	0.80	1.30
Acetone	4.90	2.70	3.40
Formic acid	5.00	5.20	3.60
Butane-2,3-dione	1.60	3.50	1.70
2-Oxopropanal	3.40	0.60	0.80
Acetic acid	7.30	6.00	3.10
1-Hydroxypropan-2-one	7.30	10.70	1.50
*sec*-Butyl acetate	1.80	0.90	2.00
Furfural	40.40	42.40	48.20
3-Furancabaldehyde	2.10	1.90	2.80
Furylmethanols	2.00	2.80	4.70
Methylfuraldehydes	9.40	13.30	17.30
Furylacetates	1.20	1.60	2.80
2,2-Dimethyl-3(2*H*)-furanone	1.40	1.70	0.90
*Furans, total*	*72.50*	*64.40*	*79.00*

**Figure 10:**
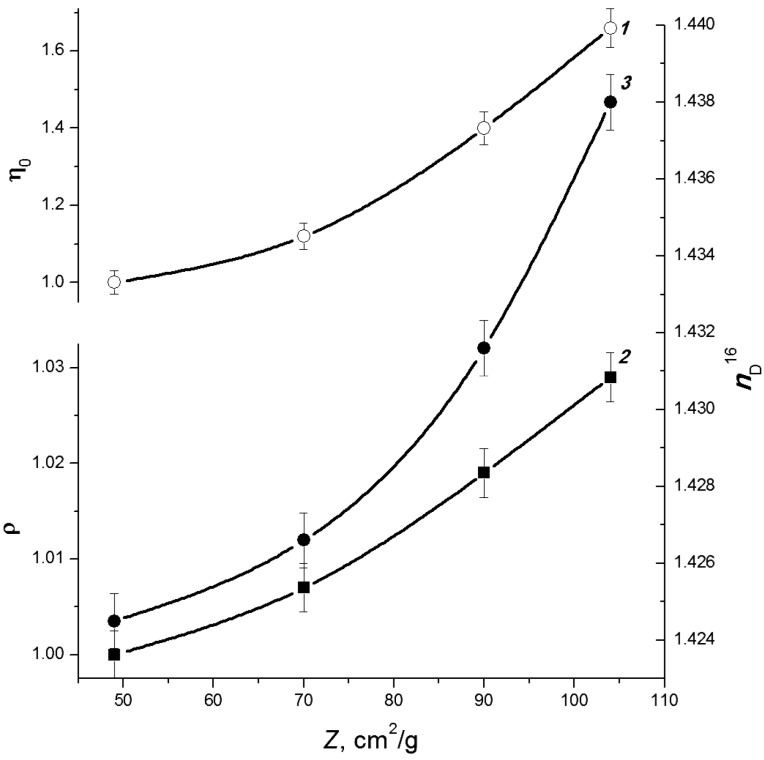
Effect of the specific surface area of cellulose C2 samples on density (*1*), viscosity (*2*) and refractive index (*3*) of condensates [45]. Here and in Figure 12, the relative changes in the density and viscosity are shown (these parameters of the sample with *Z* = 48 cm^2^/g are taken to be unity).

Grinding of cellulose C2 has practically no effect on the duration and intensity of the electron-beam distillation. The condensate yield remains unchanged, being 58% ± 1% of the cellulose weight. However, a decreasing tendency in the charcoal residue yield and an increase in the gaseous products yield of approximately 2–3 mass% was observed for more finely ground samples C2. The condensate composition also changed: the more finely ground is the starting material, the higher are the density, viscosity, average molecular mass and refractive index (Figure 10).

**Figure 11:**
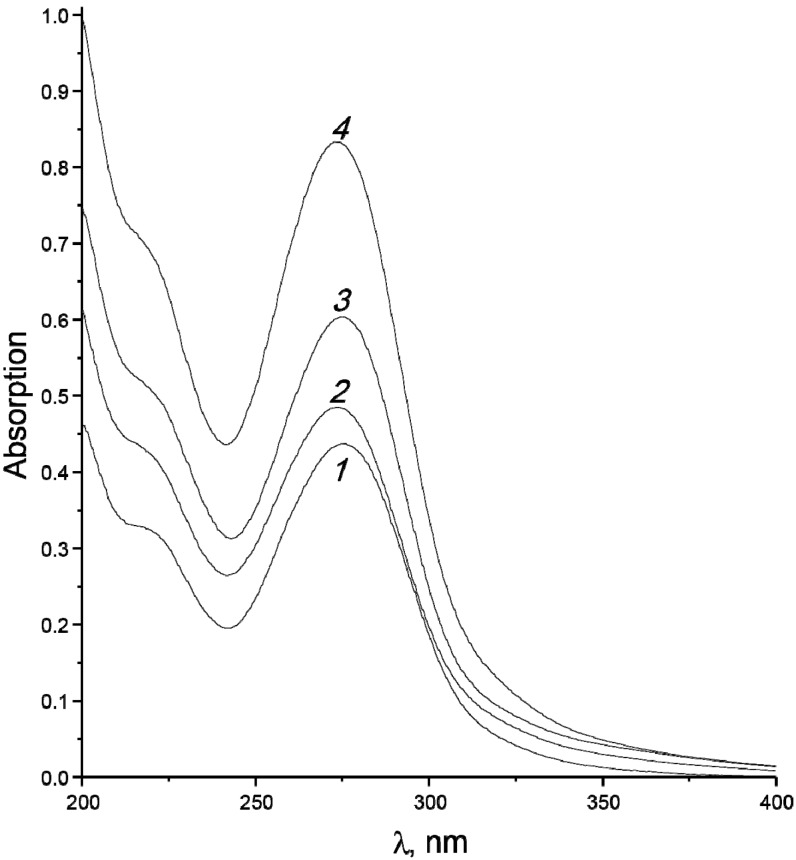
UV spectra of condensates obtained from cellulose C2 samples with specific surface areas of 49 (*1*), 70 (*2*), 104 cm^2^/g (*3*) at starting temperature of 16 °C, and also for a sample with *Z* = 104 cm^2^/g (*4*) at 230 °C [37].

As the cellulose specific surface area increases, the absorption band of the condensate at 275 nm becomes more intense (Figure 11). This band is due to absorption of furan derivatives, its increase indicates that the yields of these compounds increase with increase of dispersity of the starting material.

Thus, the following trends for condensate composition are observed with increase in the cellulose specific surface area:

- the content of the fraction of furan derivatives, which represent large fragments of glucopyranose (furancarbaldehydes, furylmethanols, furanones, *etc.*), increases;
- the content of the fraction of small glucopyranose fragments (1-hydroxyacetone, acetic and formic acids) decreases;
- the content of the fraction of heavy products (furylmethyl acetates), which are formed apparently in the secondary reactions between the major primary products, also decreases.

The products of radiolytic fragmentation of cellulose are formed both on the surface and in the bulk of the material. Migration of the fragments from the bulk to the surface (into the mobile vapour phase) takes some time, thereby the probability of a secondary radiolytic or thermal decomposition of the primary products and their reactions with other products (to give “mixed” compounds) increases. Apparently, all this changes the composition of condensates obtained from samples with different degrees of grinding.

**Figure 12:**
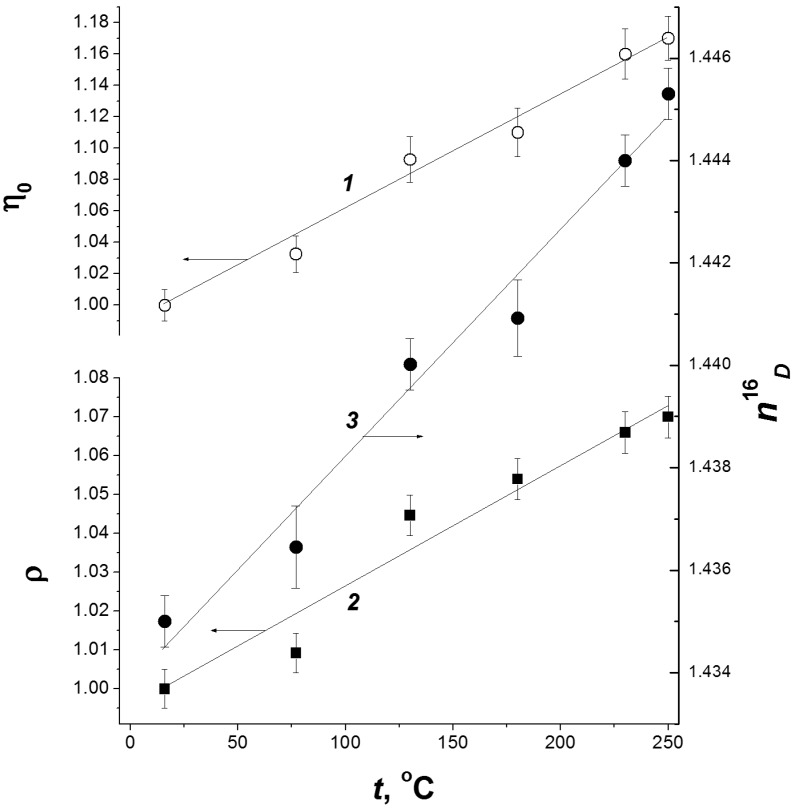
The effect of the starting temperature of radiolysis of C2 samples (*Z* = 104 cm^2^/g) on the density (*1*), viscosity (*2*) and refractive index (*3*) of condensates [37].

To estimate the contribution of pyrolytic decomposition of samples [1], the effect of cellulose preheating (before irradiation) on the electron-beam distillation at ~250 °C was studied. It was found that the change in the initial temperature has an insignificant effects on the condensate yield. According to the telemetry data, the time elapsed before vapour appeared in the heated samples was reduced slightly, but the time of completion of the distillation did not depend on the starting temperature. This fact indicates that the dynamics of cellulose decomposition are controlled by radiolysis and the radiolytic heating stages but is not determined by the temperature of the feedstock. The condensate yield was 58.0 ± 1.5 mass% when the temperature of the feedstock was varied from 16 to 250 °C. Pyrolysis of cellulose is usually initiated at ~270 °C. It may seem logical that radiation heating of hotter samples should result in faster distillation; however this is not the case.

The data shown in Figure 12 indicate that preheating favours the formation of heavier condensate: when the initial temperature of the raw material before irradiation was increased, the resulting condensate had higher density, viscosity and refractive index. The average molar mass of the condensate increases monotonically from ~87 to ~94 g/mol. The optical density at λ_max_ = 275 nm (characteristic band of furans) also increases as it is seen in Figure 11. The furan derivative fraction in the condensate grows from 60 mass% to 72 mass%, being mainly composed by furancarbaldehydes (furfural content is ~75%).

The effect of preheating is most significant in the initial steps of radiolysis: faster migration and removal of primary decomposition products and thermal decomposition of labile fragments take place. This precludes participation of the primary products in further radiolytic transformations. The sample preheating experiments reveal the predominant influence of radiolytic processes on the formation of final products of the distillation. The vigorous chain formation of carbon dioxide and other products and their release into the gas-vapour phase prevent cellulose overheating and thereby prevent its decomposition and distillation from following the unfavourable pyrogenetic mechanism.

**Figure 13:**
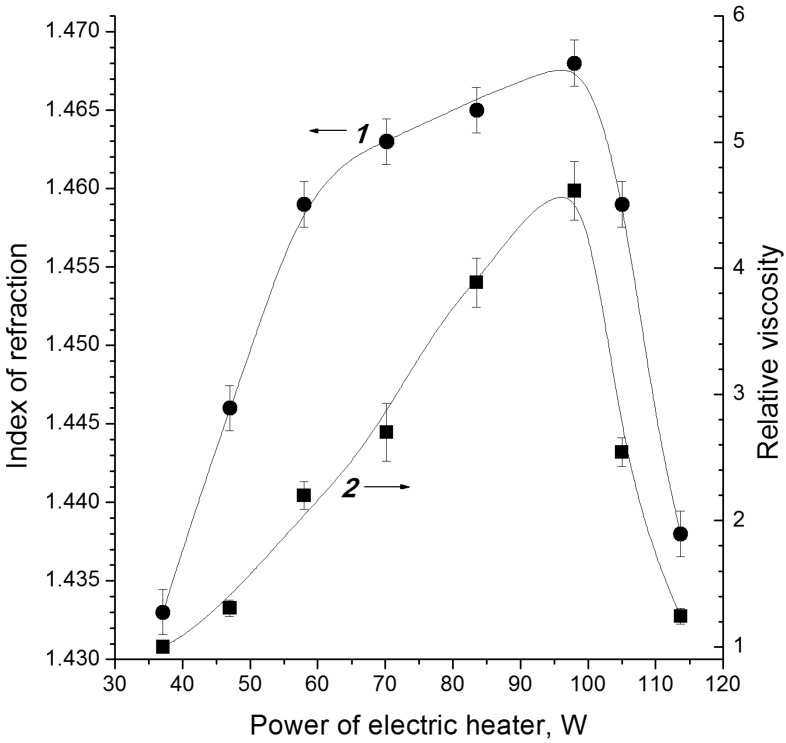
Effect of additional electric heating on refractive index (*1*) and relative viscosity (*2*) of the condensate distilled from microcrystalline cellulose at dose rate of 0.42 kGy/s and starting temperature of 80 °C [50].

As mentioned above, preheating of cellulose has no significant effect on its electron-beam distillation. Nevertheless, the supplementary heating during irradiation increases the yield of liquid products almost up to 70 mass% and decreases the yields of charcoal and gases. As shown in Figure 13, with increase in the power of a supplementary electrical heater, the condensate being distilled becomes heavier due to increase in the fraction of furan derivatives. This id observed only if the simultaneous radiation and supplementary electrical heating maintain the cellulose sample at a temperature (during distillation of low-molecular-mass products) of up to 270 °C (the onset of pyrolysis). If the supplementary heating is too intense, conventional pyrolysis (dry distillation) starts to prevail, the sample is heated fast to the pyrolysis onset temperature, the yield of the distilled condensate decreases, and water becomes predominant component in the condensate (the viscosity and the refractive index decrease, see Figure 12).

The major gaseous cellulose decomposition products formed upon electron-beam distillation are CO_2_, H_2_ and CO. Equimolar formation of carbon dioxide and hydrogen is observed, while the CO yield is lower by an order of magnitude. Stoichiometrically, the fraction of the -O-C-O- groups (CO_2_ precursor) in the glucopyranose C_6_H_10_O_5_ unit is ~27 mass% of the starting cellulose dry weight. At the same time, the experimentally observed CO_2_+CO total yield from cotton cellulose is almost twice as low. Therefore there is deficiency between the amounts of eliminated carbon dioxide and decomposed glucopyranose units.

Recall that if cellulose is irradiated at temperatures below the pyrolysis temperature range (≤190 °C), the yields of compounds containing carbonyl groups and CO_2_ coincide within the experimental error (see Section 3) with the yield of polymeric chain decomposition. This indicates that each cellulose chain cleavage event is accompanied by decomposition of a glucopyranose unit to afford CO_2_ and a carbonyl group. In the course of electron-beam distillation, the glucopyranose ring decomposition provides a lower yield of CO_2_. Thus, under these conditions, decomposition of glucopyranose units, resulting mainly in formation of furan derivatives, is most likely not always accompanied by elimination of CO_2_.

**Figure 14:**
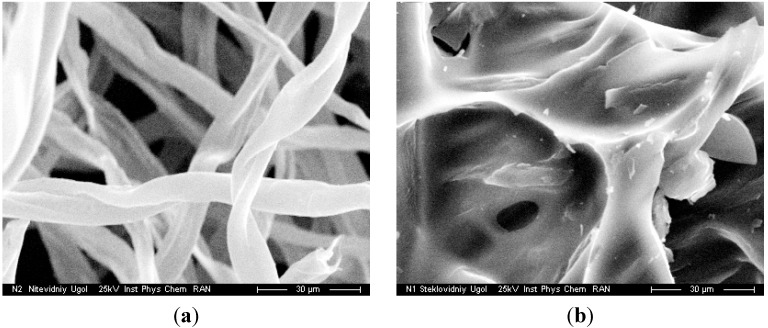
Photomicrographs of two structural modifications of charcoal (the scale is 105 × 80 μm) [37].

The charcoal residue from the C1, C2 and C3 cellulose samples consists of two fractions, namely, long fibres (their structure is similar to that of initial cellulose; the atomic ratio C:O = 8.4; Figure 14a) and foam inclusions (as drops and films; the atomic ratio C:O = 6.4; see Figure 14b). The oxygen depleted components could be rather important precursors of charcoal remaining at the end of distillation and forming the first fraction. A part of the charcoal can be formed in condensation reactions of decomposition products resulting in high-boiling compounds, whose evaporation from the reaction zone is difficult. The second fraction occurs on the charcoal surface and is obviously formed in the condensation. Particularly, condensation processes are typical of furfural (the empirical formula is C_5_H_4_O_2_) and some of its derivatives under the dry distillation conditions, for example, by Equation (9) [1,51]:

2C_5_H_4_O_2_ → C_10_H_6_O_3_ + H_2_O (9)

As shown [51] furfural quickly decomposes in an acid medium yielding formic acid and humic compounds (50 g of formic acid and 41.5 g of humic compounds are formed from 100 g of furfural). The formation of high-molecular-mass products upon reactions of furfural with other intermediates could also promote an additional carbonization of the initial material. Evidently, fast removal of vapours from the irradiation area is a major way to decrease the yield of high-molecular-mass products and to minimize the loss of furfural homologues [51].

In practice, furfural can be formed upon acid hydrolysis of pentosanes, which belong to easily hydrolyzable vegetable hemicelluloses of the general formula C_5_H_10_O_5_ [51]:

C_5_H_10_O_5_ − 3H_2_O → C_5_H_4_O_2_ (10)

In contrast to hemicelluloses, hydrolysis and pyrolysis of cellulose has a low probability of formation of furfural and furylmethanol [1,51]. Moreover, as we pointed out above, no formation of furan derivatives was observed upon irradiation of cellulose at temperatures of ≤190 °C. Therefore, the high yield of furans observed in the electron-beam distillation is due to the specificity of the cellulose decomposition mechanism involved in this unusual radiation-thermal process.

Thus, the main result of the electron-beam distillation of cellulose of various types is formation of liquid organic condensate. Furylmethanol, furfural and their derivatives are the major liquid products. Grinding and preheating the starting material promote an increase of the furan fraction in the condensate. The electron-beam distillation process could be a key to development of promising methods of production of furans from widespread plant materials and their processing wastes.

### 3.3. The Mechanism of Radiation-Thermal Transformations

The procedure of electron-beam distillation of cellulose could be conditionally divided into two phases [52]. The first phase is radiolytic decomposition independent of temperature giving radical fragments with unpaired electrons located at the chain end. This phase smoothly transforms into the subsequent phase, the high-temperature (220–250 °C) decomposition of such fragments. At the dose rate of the electron-beam treatment of 2–3 kGy/s, vapours start to condense in the condenser ~3 min after the beginning of irradiation (conditionally, the first phase proceeds during this period), and the vapour generation is finished 6 min later (the second phase is completed).

The vast majority of low-molecular-mass products of the distillation have 1 to 6 carbon atoms. Analysis of their composition shows that their common precursor is the glucopyranose unit (C_6_H_10_O_5_). It is important to emphasize that the major products of the high-temperature radiation decomposition are furfural (or furylmethanol) and other furan derivatives. This set of products is untypical of both cellulose radiolysis at moderate temperatures and cellulose pyrolysis without irradiation. Therefore, a specific mechanism of transformations operates during the electron-beam distillation of cellulose. Below we consider and discuss this mechanism.

As we have already stated, at moderate temperatures (≤190 °C), during the first phase, radiation initiates the decomposition of cellulose with formation of oligosaccharides and radicals. The primary radicals are produced according to reactions (2)–(7) upon C-H bond cleavage mainly in positions 1 and 4 of glucopyranose. These bonds are unstable because of mismatch of electron configurations of the radical centre and the initial glucopyranose unit (see above) [2,3,10], which is secured by hydrogen bonds in the rigid framework of cellulose. The radical transforms into the stable configuration with the flat *sp^2^* hybridization of the carbon atom upon decomposition of the glucopyranose unit and cleavage of the polymer chain. As a result, the stable radical states in the irradiated cellulose are located at the terminal fragments of the polymer chain (see Scheme 1).

The second phase is decomposition of cellulose fragments into low-molecular-mass compounds under high-temperature radiolysis (radiation pyrolysis) conditions in the range of 220–250 °C. Processes that occur under these conditions are determined by the intensive dehydration of cellulose and, especially, its shorter fragments formed upon decomposition. In the course of dehydration of a polysaccharide, consecutive elimination of water molecules may finally result in carbonization of the material. Glucopyranose undergoes deep chemical transformations and structural reorganization, *viz*., double bonds appear and conformations of polymer units change.

**Scheme 3:**
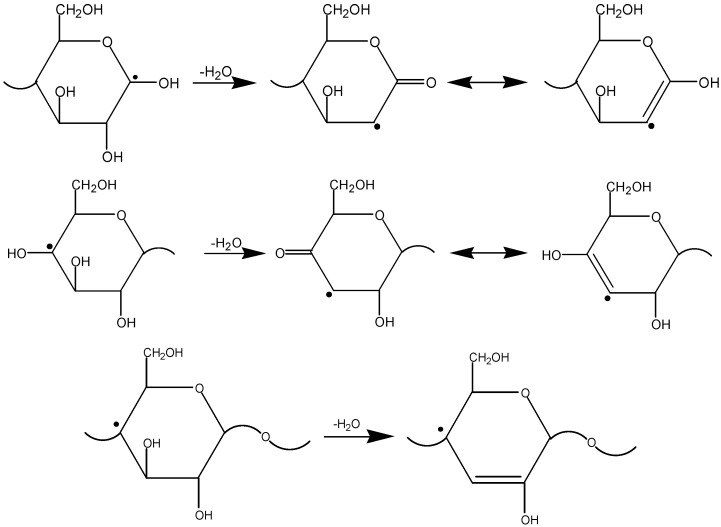
Thermally stimulated dehydration of the terminal radicals at the C(1) and C(4).

Note that dehydration of radicals starts at substantially lower temperatures than that of molecules. For instance, transformations of terminal radicals in positions C(1) and C(4) in the irradiated cellulose into allyl type radicals is already observed at ~100 °C and above, whereas water molecule elimination during the cellulose pyrolysis starts above 200 °C [53,54]. During the thermally stimulated dehydration, the terminal radicals at the C(1) and C(4) acquire an allylic structure as shown in Scheme 3.

This process was detected using EPR spectroscopy [10,19,55]. In turn, conjugation of the C=C bonds destabilizes the C-C and C-O bonds, thus facilitating the opening of the glucopyranose ring and/or elimination of some fragments from it.

**Scheme 4:**
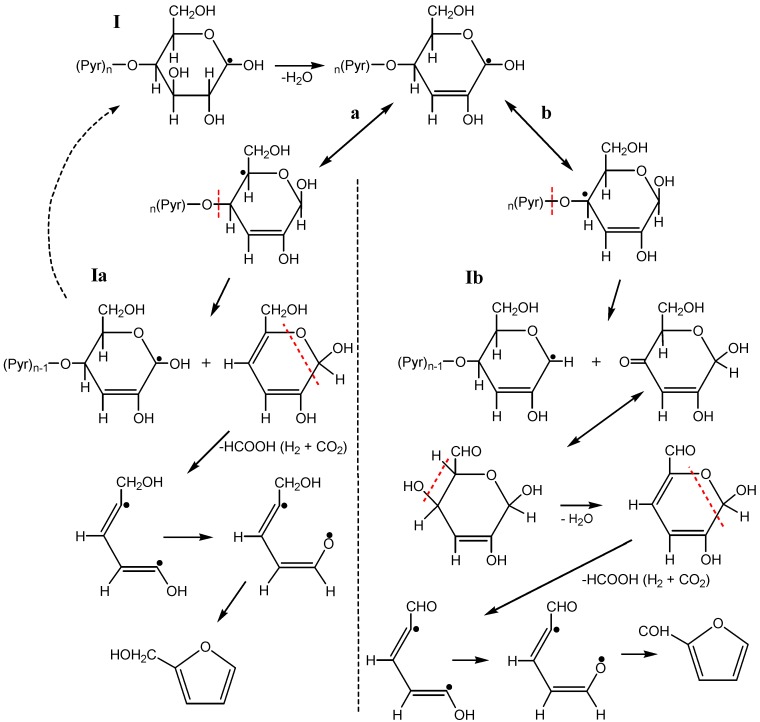
The furan formation from a C(1) macroradical.

The radiation-thermal process implements limited glucopyranose ring decomposition pathways that result in formation of a dominating product and a reduction in the assortment of byproducts. The important role belongs to so-called β-cleavage when the weakest bond, located in the β-position with respect to the unpaired electron localization site, undergoes homolytic cleavage. In the course of homolytic cleavage the larger substituent is eliminated easier in the form of a radical as the long radical can be stabilized easier via one electron delocalization. The respective change of the unpaired electron localization site in a radical can be a consequence of hydrogen, vinyl, acyl and acyloxy group transfers. Scheme 4 shows the furan formation from a C(1) macroradical, in agreement with the observed sequence of cellulose decomposition.

The macroradical (**I**) undergoes a thermostimulated decomposition resulting in chain formation of furanmethanol (via radical **Ia**) or furfural formation (via radical **Ib**). Radical **Ia** has the same structure as radical **I** but is shorter (by one pyranose unit). Radical **Ib** has another structure which however does not exclude chain formation of furfural. The high observed yield of furfural formation can be caused also by thermal dehydrogenation of furanmethanol (or of its nearest predecessors). The possibility of furan formation can be assumed too in case of C(4) macroradical on the basis of similar regularity controlling thermostimulated dehydration and decarbonylation [52].

The “catalyzing” effect of macroradicals can be revealed by the accelerated dehydration at lower temperatures in comparison with conventional pyrolysis. The arising conjugated bond system is conducive to transfer of the radical centers and hydrogen atoms along the polymer chain. As a result of the occurring reactions the macroradical center is transferred from the terminal pyranose unit to the penultimate one, and the terminal unit is eliminated to form a furan fragment (furanmethanol or furfural). The presence of double bonds (*sp*^2^-hybridization and planar structure) side by side with -CH(OH)- or -CH(H)- groups (*sp*^3^-hybridization and tetrahedral structure) provides the structural stress in the intermediate, promoting the decomposition. Formic acid and formaldehyde are reactive byproducts susceptible to condensation reactions typical of carbonyl and carboxylic organic compounds.

The chain radiation-thermal decomposition propagates into all parts of the cellulose sample. The close packing of surrounding molecules can interfere with the removal of arising radical and molecular fragments. In these conditions allyl radicals can be transformed to polyene type radicals [19,55], which are more stable and play a role as charcoal precursors. We discovered the interesting fact that electron-beam distillation of cellobiose and cyclodextrin as low-molecular-weight analogues of cellulose produces only trace amount of furan derivatives. This fact finds the explanation within the limits of the above described chain transformations mechanism. There are no conditions for propagation of chain intramolecular decomposition in a cellobiose consisting of two pyranose units whereas some steps of such decomposition are probable in cyclodextrins. Thus chain transfer to the other molecules of the condensed phase, is probably complicated and slower than radical recombination processes.

The predominant formation of furan derivatives and CO_2_ deficiency indicating that decomposition of the glucopyranose unit is accompanied by elimination of some other compound and a substantially lower yield of water in the polysaccharide decomposition confirm this mechanism of radiation-thermal transformations of cellulose during the electron-beam distillation. Particularly, in the course of the electron-beam distillation of cellulose, according to Scheme 3, two water molecules are eliminated due to dehydration, while approximately five water molecules should have formed upon predominant decomposition of glucopyranose unit to charcoal in the course of dry distillation. Indeed, water is formed in approximately such a ratio in the processes under comparison. This also clarifies why cellulose preheating has no significant effect on the electron-beam distillation process. Indeed, according to the stated mechanism, a stage consisting in the decomposition of cellulose with formation of fragments with terminal radical centers should precede its radiation-thermal decomposition.

**Scheme 5:**
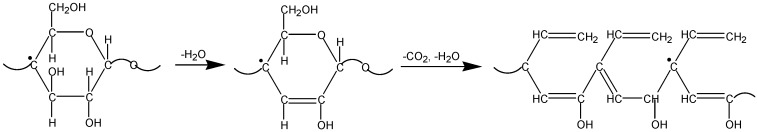
Formation of polyene type radical from allyl radical.

The chain radiation-thermal decomposition of cellulose propagates through the entire material. The close packing of surrounding molecules may prevent the removal of formed radical and molecular fragments. Allyl radicals are capable of rearranging into polyene type radicals [19], which are more stable and can be precursors of charcoal (Scheme 5).

At low dose rates (~0.15 kGy/s) insufficient for efficient heating and initiation of distillation, cellulose remains solid and becomes yellow. The yields of condensate, in which water prevails, and coal upon post-radiation (~500 kGy dose) dry distillation are ~47 mass% and 32 mass%, respectively. The substantial reduction of the yield of the condensate as compared with the electron-beam distillation indicates the importance of the reactions occurring during radiolysis. Only thermal degradation of stable molecular fragments of cellulose with reduced degree of polymerization occurs in the post-radiation dry distillation. This fact confirms the conclusion that the intermediates participating in radiation-thermal cellulose decomposition differ from those in conventional pyrolysis. Accordingly, the yields of the electron-beam distillation products differ from the yields observed in post-radiation distillation.

Radiolytic decomposition of cellulose as a component of wood (*i.e.*, without isolation) is less efficient than decomposition of isolated and purified cellulose fibers [56]. Evidently, protection of cellulose against radiation in the wood bulk is due to excitation energy transfer from cellulose to lignin containing more stable aromatic fragments [1,57].

**Figure 15:**
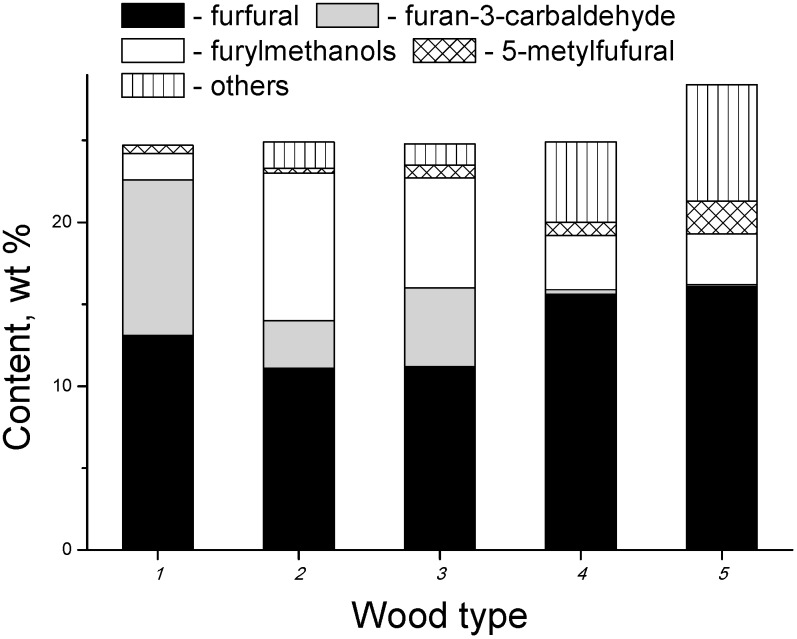
Yield and composition of furans fraction of in the condensates obtained upon electron-beam distillation of various wood (*Z* = 90 ± 5 cm^2^/g, bulk density is 150 g/dm^3^): *1*—birch, *2*—aspen, *3*—alder, *4*—spruce, and *5*—pine [58].

The radiation-thermal decomposition of both softwood and hardwood produces a large furan fraction (see Figure 15). As noted above, the major source of furans is cellulose. Furancarbaldehydes prevail among furan derivatives. The electron-beam heating of hardwood results in the formation of both furan-3-carbaldehyde and furan-2-carbaldehyde (furfural). Furfural is a dominant compound of the furan-carbaldehyde series upon decomposition of softwood. The condensates from aspen and alder have a larger fraction of furylmethanols, whereas the pine condensate is relatively enriched in 5-methylfurfural. The condensates obtained from softwood contain more diverse furans than hardwood condensates. For instance, the pine condensate, in addition to aldehydes, contains furan, 2-methylfuran, 2,5-dihydro-furan, 2,5-dimethylfuran, 2,5-dihydro-3-methylfuran and 5-methylfuran-2(3*H*)-one. The wide variety and the high total yield of furans obtained from softwood may be due to lower fragmentation capability of cellulose in the presence of aromatic compounds.

## 4. Advanced Applications

Cellulose, which chemically is a polymer with repeating glucose units, containing a lot of carbon and hydrogen, has generated great interest as a potential renewable raw material to produce organic reagents and fuel [59,60,61,62]. Nowadays the cellulose fraction of a biomass can be considered *per se* as an alternative to steadily exhausting reserves of natural oil, gas and coal. Probably, already in this century, the advanced processing of biomass may become the important constituent of feedstock delivery into the energy and chemical industries [63,64,65,66]. For economic and environmental reasons, inedible cellulose-containing raw materials would be the most suitable for large-scale industrial conversion. Significant amounts of such raw materials are formed by wood processing (up to half of used forest products) and by agriculture (straw, corncobs, husk and many others) [67,68,69,70]. The creation of advanced technologies (direct, productive and cheap) for cellulose conversion is the important problem.

Cellulose is the main component (50–100 wt %) of renewable vegetative biomass [1]. Nowadays the biochemical treatment methods based on enzymatic hydrolysis of cellulose have the broadest dissemination and high potential to produce power gas, wood charcoal, various kinds of liquid bio-fuels, and some other products [71,72,73,74,75]. Biochemical processing of natural polymers is being upgraded continuously, and accordingly becoming proclaimed more and more as a new production technology for bio-ethanol and other fuel oxygenates [76,77,78,79,80,81,82,83,84,85,86].

Undoubtedly, other techniques, first of all, direct methods of cellulose conversion to valuable products deserve steadfast attention also, in particular, the abovementioned method of electron-beam distillation. As follows from the presented material, the use of high-energy accelerated electrons makes it possible to implement a direct single-stage conversion of cellulose biomass into liquid condensate. In comparison with pyrolysis, electron-beam distillation has several advantages: it hampers water formation, halves the accumulation of charcoal and increases ~6-fold the yield of liquid organic products [13,87,88,89]. Such high performance of the process is related to the specific mechanism operating upon simultaneous action of ionizing radiation and heating.

Liquid products of electron-beam distillation cannot be directly used as fuel. The presence of unsaturated bonds and active functional groups gives rise to undesirable chemical reactions (dehydration, polycondensation and so on) between condensate components. Alkanes, benzenes, aliphatic alcohols, ethers and esters are usually considered as suitable liquid fuel components [90]. Tetrahydrofuran derivatives are also considered as satisfactory components of high-octane motor fuel [90,91].

The electron-beam treatment of cellulose can be carried out in a reactor filled with hydrogen or light alkanes as hydrogenation or/and alkylation agent. Gaseous alkane also participates in the process, being a precursor of liquid alkanes. The liquid alkane fraction in the condensates is mainly composed of highly-branched isomers. On the other hand, some alcohols and ethers, which are not typical of electron-beam distillation of cellulose feedstocks, are observed among the oxygen-containing compounds. Obviously, these compounds are formed upon radiolytic transformations of the alkane used as the carrier gas. Note that the procedure of electron-beam distillation of cellulose in a light alkane medium provides a fixation yield of gaseous alkane an order of magnitude higher than radiolysis of the gas without addition of cellulose feedstock [13,92]. This may be based on transfer of radical, charge and energy between intermediates originating from cellulose and the alkane. As a consequence, more active participation of the gas in the formation of liquid alkanes, alcohols and ethers take place.

Comprehensive studies show that hydrogenation and/or alkylation of organic vapours obtained in electron-beam distillation of cellulose-containing biomass give stable mixtures, which can be used as fuels (engine, diesel, rocket or boiler fuel) [93,94].

The charcoal yield upon conventional dry distillation is 38 mass%. It is close to the theoretical yield (45 mass%) expected of dehydration mechanism of glucopyranose decomposition according to the following equation:

C_6_H_10_O_5_ → 6C + 5H_2_O (11)

Thus, only ~7 mass% of carbon is consumed for formation of organic compounds and gaseous CO_2_ and CO.

The charcoal yield in electron-beam distillation of cellulose [45,48,95,96] does not exceed 20 mass%. Therefore, the degree of carbon conversion to liquid organic compounds amounts to ~25 mass%, which is a consequence of glucopyranose unit decomposition mainly according to the reaction:

C_6_H_10_O_5_ → C_5_H_4_O_2_ + HCHO + 2H_2_O (12)

Optimization of radiation-thermal decomposition of lignocellulose material could result in the decrease in charcoal yield and, therefore, in the increase in the yield of the desired liquid condensate. The loss of carbon at the first, “incubatory” step of radiation destruction giving carbon dioxide is unavoidable. Estimates show that the carbon loss at this step does not exceed 3 mass% ± 5 mass%. Therefore, there is a substantial margin for process optimization. In our opinion, to optimize the process, it is necessary: (i) to reduce the temperature of distillation; (ii) to achieve the most complete removal of products of primary decomposition from the irradiation area; (iii) to use gases and additives for activation of decomposition of cellulose feedstock; (iv) to optimize irradiation conditions; (v) to enhance the degree of dispersion of the raw material, *etc.*

## 5. Experimental Section

**Figure 16:**
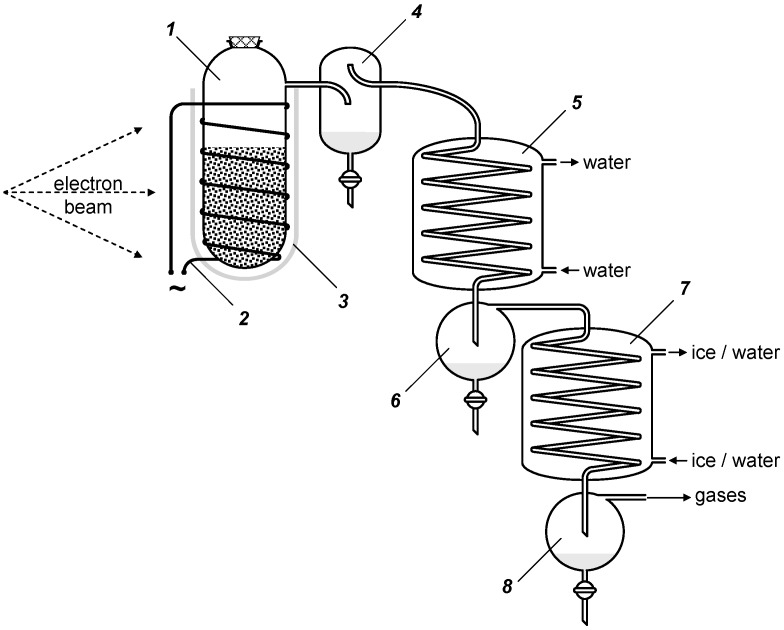
Schematic of the laboratory installation.

Cotton cellulose (fibers, GOST 5556-81), pine sulfate unbleached cellulose EKB-1 (cardboard, GOST 12765-88), pine sulfite bleached cellulose (cardboard, GOST 3914-89), and microcrystalline cellulose (powder, from Alfa Aesar, Lancashire, United Kingdom) were studied. Cardboard (sheets of 1 mm thickness) were used after cutting (to ~2 × 2 mm). The influence of the type and initial temperature was studied for the samples with the specific surface *S* = 104 ± 4 cm^2^·g^−1^. Lower specific surface of unbleached cellulose was obtained by additional cutting of the cardboard. Cellulose samples were dried up at 107 °C and deaerated before investigation. Samples were irradiated by the accelerated electrons (UELB-10-10T linac; beam energy, 8 MeV; pulse duration, 6 μs; pulse repetition frequency, 300 Hz; average beam current, 800 μA; scan width, 245 mm; and scan frequency, 1 Hz) at air temperature 17 ± 2 °С. Distillation was carried out in the laboratory installation shown in Figure 16. Samples were irradiated in 100 mL cylindrical quartz vessels ***1*** (40 mm diameter) at atmospheric pressure without air access. The reaction vessels were filled by 60% at an average packing density of 0.15 g·mL^−1^. The uniformity of absorbed dose in bulk of cellulose was provided due to low density of samples and high energy of incident electrons (range was ~250 mm). Reaction vessels have been supplied by an electric heating coil ***2*** and heat insulator ***3***. Three consecutive coolers-condensers ***4***, ***5*** and ***7*** (cooled accordingly by air at 17 ± 2 °C, by water at 15 ± 2 °C and by ice-water mix at ~0 °C) were located outside radiation area and were adjusted for roughing-out and condensation of light fragmentation products. Liquid fractions go into receiving containers ***4***, ***6*** and ***8***. Gaseous products were liberated by inertial separation in reception container ***8*** and were evacuated to ventilation.

Film dosimetry with a phenazine dye-doped copolymer standard reference material SO PD(F)R-5/50 [GSO (Certified Reference Material) no. 7875–2000] was used. Dose rate adjusted by distance change between a reaction vessel ***1*** and accelerator beam window at invariable beam parameters (800 μA, 8 MeV). Beam diameter was more or equal to diameter of a reaction vessel ***1***. The electron beam was being scanned along a vertical axis of reaction vessel ***1*** within angle ± 17°. Under these conditions the reactor height was always less than the scanning beam amplitude. Accordingly, the electron beam deviated periodically above or below a reactor, giving a discontinuous irradiation of the sample.

In M1 mode the vessel containing the irradiated sample (at 0.02 kGy/s) was seated in a 200 Watt muffle furnace (pre-warmed to 200 °С) and was heated-up until termination of pyrogenic distillation of the sample.

Indexes of refraction and density of distilled-off condensates were measured by means of an IRF-454BM refractometer and VIP-2M densitometer. A gas chromatograph/mass spectrometer (Q-Mass Perkin-Elmer AutoSystem XL; helium as carrier gas; 60-m glass capillary column of 0.25 μm inner diameter; NIST library of mass spectra) was used for primary analysis of light products.

## 6. Conclusions

It has been determined that decomposition of cellulose under electron-beam distillation conditions occurs by a pathway distinct from those typical of pyrolysis, room-temperature radiolysis, acid and enzymatic hydrolysis, and others. The powerful electron beam promptly initiates uniform three-dimensional warming up of cellulose as well as formation of macroradicals, thereby creating unique conditions for radiation-thermal conversions of the biopolymer. The distinctiveness of this approach is that furfural and other furan derivatives are originated as the prevailing products of cellulose decomposition. As an emotional stimulus, the pronounced furfural-intrinsic odor of the newly-baked rye bread accompanies this new method of cellulose transformation. This odor is sharply distinct from the typical smell of the tar peculiar to pyrogenous distillation of cellulose-containing materials.

The above described electron-beam conversion of cellulose is a method ready for optimization. For example, feedstock heating can be carried out by low-power electron beam in a combination with a conventional auxiliary heater to reduce the cost of electron-beam processing [50,52]. As another example, radiation-thermal conversion can be performed in a hydrogen or gaseous alkanes medium to produce hydrogenated and/or alkylated furan derivatives [50,52].

Recently the US Department of Energy has published a list of key products which are especially desirable to be originated from vegetative biomass [90]. Furans occupy an important place in this list. Along with applications in fuel [91] and polymer production, they could be considered as potential substitutes of oilstock for obtaining various other valuable products. However ways for the large-scale industrial production of furans have not been revealed till now. Apparently, electron-beam treatment of cellulose differs by the high yield of furans and thereby can become such a direct method—ecologically and economically sound—to convert the renewable vegetative biomass for the purpose of subsequent production of fuels and as a feedstock for organic synthesis.
